# Trace elements and the thyroid

**DOI:** 10.3389/fendo.2022.904889

**Published:** 2022-10-24

**Authors:** Qing Zhou, Shuai Xue, Li Zhang, Guang Chen

**Affiliations:** ^1^Department of Thyroid Surgery, General Surgery Center, The Hospital of Jilin University, Changchun, China; ^2^Department of Nephrology, The Hospital of Jilin University, Changchun, China

**Keywords:** trace elements, thyroid, hyperthyroidism, hypothyroidism, autoimmune thyroid diseases

## Abstract

Trace elements, such as iodine and selenium (Se), are vital to human health and play an essential role in metabolism. They are also important to thyroid metabolism and function, and correlate with thyroid autoimmunity and tumors. Other minerals such as iron (Ir), lithium (Li), copper (Co), zinc (Zn), manganese (Mn), magnesium (Mg), cadmium (Cd), and molybdenum (Mo), may related to thyroid function and disease. Normal thyroid function depends on a variety of trace elements for thyroid hormone synthesis and metabolism. These trace elements interact with each other and are in a dynamic balance. However, this balance may be disturbed by the excess or deficiency of one or more elements, leading to abnormal thyroid function and the promotion of autoimmune thyroid diseases and thyroid tumors.The relationship between trace elements and thyroid disorders is still unclear, and further research is needed to clarify this issue and improve our understanding of how trace elements mediate thyroid function and metabolism. This paper systematically reviewed recently published literature on the relationship between various trace elements and thyroid function to provide a preliminary theoretical basis for future research.

## Introduction

The thyroid gland plays a key role in homeostasis, growth and development, and normal reproductive, nervous, and cardiovascular system function. Thyroid function is regulated by the hypothalamic-pituitary-thyroid axis and mediated by thyrotropin-releasing hormone (TRH), thyroid-stimulating hormone (TSH), triiodothyronine (T3), and thyroxine (T4). Thyroid disease is a common endocrine disorder increasing in prevalence, and the etiology of thyroid disease is gaining more attention. Although research is inconclusive, the relationship between trace elements and thyroid diseases are investigated.

Trace elements are essential for human survival and many physiological processes, including those of the thyroid gland, where the concentration of many trace elements is higher than that of other tissues (1). The thyroid affects trace element metabolism, and the level of trace elements also affects normal thyroid metabolism and function (2). A change in trace element concentration will affect the endocrine system and other body systems, causing thyroid dysfunction, including hyperthyroidism, hypothyroidism, autoimmune thyroid disease (Graves’ disease and Hashimoto’s thyroiditis), thyroid cancer, and other system diseases.

In this review, we will evaluate the relationship between trace elements and thyroid function and discuss the interaction between various trace elements and thyroid diseases. We hope to provide a theoretical basis for future research.

### Iodine

Iodine is an essential trace element that is a component of T4 and T3. Inadequate iodine intake can impair thyroid function and lead to goiters, cognitive-developmental disorders, and congenital abnormalities, collectively known as iodine deficiency disorders. Inadequate iodine intake is closely related to nodular goiters (3). In mild iodine deficiency, the thyroid gland can adapt and maintain thyroid hormone production within normal limits. However, long-term adaptation of iodine deficiency leads to follicular cell proliferation, autonomous thyroid growth and disfunction (4).

Iodine deficiency causes nodular goiter as follows. Iodine is an antioxidant that inhibits the production of hydrogen peroxide(H2O2) which is the major source of free radicals or reactive oxygen species(ROS) (5). Excessive production of H2O2 and ROS caused by iodine deficiency could lead to increased mutations in genes associated with thyroid cell growth. This may lead to the appearance of autonomous thyroid cell cloning, which promotes thyroid hormone production and decreases the level of TSH (4, 6). This also explains the phenomenon that TSH levels do not increase in people with iodine deficiency, but rather decrease. Compared to people with normal or excessive iodine intake, people with chronic mild-to-moderate iodine deficiency have a higher prevalence of hyperthyroidism and lower TSH levels. We expect that iodine deficient individuals do not produce enough thyroid hormone and having rising TSH levels, but the opposite occurs. Iodine deficiency reportedly increases the incidence of thyroid nodules, followed by an increase in hyperthyroidism (3, 6).

Iodine intake is also linked to thyroid function. Severe iodine deficiency is responsible for hypothyroidism due to a lack of the substrate for the synthesis of thyroid hormone. As discussed previously, chronic mild-to-moderate iodine deficiency is associated with decreased TSH and hyperthyroidism. When iodine is deficiency, the thyroid gland is able to maintain normal hormone levels at the expense of autonomous growth and hyperactivity of thyroid follicular cells (4). Wolf-Chaikoff effect defines a large amount of iodine causes hypothyroidism. This is due to a transient blockade of the iodine organification resulting from the downregulation of sodium iodide symporter(NIS), reducing the transportation of iodine into thyroid cells, resulting deficiency of hormone synthesis and low thyroid hormone function (5). There is an increased risk of subclinical hypothyroidism caused by increased iodine intake, especially in those with positive thyroid antibody (7). This may be related to primary autoimmune thyroid disease. According to research, high oral doses of iodine in patients with iodine deficiency can accelerate autoantibody production. When patients with Hashimoto’s thyroiditis are exposed to high levels of iodine, the risk of hypothyroidism increases (3). It is unclear if this situation is temporary, and further study is warranted.

The relationship between iodine intake and thyroid autoimmunity is complex and remains a debate. The presence of thyroid antibodies is an important signal of thyroid autoimmunity and is closely related to the severity of thyroid lymphocyte infiltration (8). Some research suggests that high iodine intake can cause an elevated thyroid antibody concentration and that large doses of oral iodine can accelerate thyroid antibody production in iodine-deficient patients (9, 10). The increase of circulating thyroid antibodies with stable, high iodine intake is not particularly common, but a sudden increase of iodine intake may induce thyroid autoimmunity. When iodine intake is suddenly increased in iodine deficient people, thyroid autoimmune reaction will be further aggravated. People with low iodine intake reportedly have a lower prevalence of circulating thyroid antibodies while those with high iodine intake have a higher prevalence of circulating thyroid antibodies (4). However, other studies have not found an association between iodine intake and high antibody concentration (11, 12). After the worldwide implementation of mandatory or voluntary iodine fortification programs (IFP), recent studies have clearly demonstrated that the recommended iodine intake (the median iodine concentration ≤ 300ug/L) can largely reduce the risks on thyroid autoimmunity. The studies also concluded that the increase on thyroid autoimmune diseases did not mean restriction of iodine intake, the other factors affecting the increased prevalence of autoimmune disease should be considered (13). The relationship between iodine intake and the presence of circulating thyroid antibodies is more complex than is currently understood.

Some animal experiments and epidemiological investigations have demonstrated a relationship between iodine intake and thyroid cancer (14–16). A retrospective study involving 1170 patients showed that in areas with adequate iodine intake, both extremely low and high iodine intake are associated with an increased risk of thyroid cancer (17). A study before and after iodine supplementation in areas with previous iodine deficiency concluded that iodine supplementation may increase the incidence of papillary thyroid cancer(PTC) (18). The likely cause is that excessive iodine can induce toxic effects on the thyroid (5). As mentioned earlier, thyroid hormone synthesis requires high concentrations of H2O2 and iodine, H2O2 is the major source of free radicals or ROS, that cause potential damage to thyroid follicular cells. Iodine is an antioxidant that inhibits the production of H2O2. Thus, low level of iodine has no ability to inhibit H2O2 or ROS, resulting in DNA damage and mutations. It has also been shown that iodine activates mitochondria, reducing the expression of anti-apoptotic proteins and increasing the expression of p21, selectively inducing apoptosis of cancer cells (5).

Appropriate iodine intake can significantly reduce the incidence of thyrotoxicosis without increasing the incidence of persistent clinical hypothyroidism (6). Increased iodine intake in cases of iodine deficiency can reduce the prevalence of goiter, adult thyroid autonomous nodules, and thyrotoxicosis, improve intelligence in children, and reduce the risk of thyroid cancer. The relationship between a population’s iodine intake and the occurrence of thyroid disease is U-shaped, as both iodine deficiency and high iodine status are risk factors for thyroid diseases. But the threshold value of iodine excess or deficiency is equivocal and does not cover all part of the world and all groups (8, 19). Iodine intake should be regulated with great care.

### Selenium

Selenium (Se) is an important element in thyroid hormone biosynthesis and metabolism (20). Thyroid tissue has the highest Se concentration (21). Se exerts its biological function through selenoproteins, the main classes of selenoproteins are glutathione peroxidase(GPX), iodothyronine deiodinase(DIO), thioredoxin reductase(TXNRD), selenoprotein P(SEPP), selenoprotein K(SELK), etc. They are involved in many diverse biological processes, including DNA synthesis, oxidoreductions, antioxidant defence, thyroid hormone metabolism, immune responses and so on (22, 23). Se deficiency decreases the ability of T4 to transform into T3 (24). Lower serum Se levels (Se deficiency) are associated with newly diagnosed Graves’ disease and autoimmune hypothyroidism (25).

Although iodine is a major factor in the multiple etiologies of thyroid disease and is a major determinant of thyroid size, experts have proposed that Se also affects thyroid size (20, 26). During the biosynthesis and storage of thyroid hormones, the normal function of thyroid cells and vascular follicular units requires adequate Se intake. Iodine status is a major driver of changes in thyroid size in people with iodine deficiency. In iodine-rich individuals, the effect of Se on thyroid size was more pronounced than that of iodine deficient. A large intervention study has shown that inadequate Se intake is associated with increased thyroid volume in women, but not in men (27), which adds the complexity to clinical use of Se. Determining the optimal Se dosage and future research should take gender into account.

An observational study on the relationship between Se and Graves’ disease found that serum Se concentration in patients in remission was higher than that in relapsed patients (28), while serum Se concentration in newly diagnosed patients was lower than that in the control group (25). There is a complex relationship between Se concentration and Graves’ ophthalmopathy. The serum Se concentration of patients with Graves’ ophthalmopathy is lower than that of healthy subjects (29), which indicates that Se deficiency may be an independent risk factor for this condition. However, in a separate study, no significant association was found between Se level and the severity or activity of Graves’ ophthalmopathy (30). A 2019 paper reported that Se deficiency increases the risk of hyperthyroidism in both Graves’ disease and nodular goiter, but Se supplementation does not affect TSH receptor autoantibody levels and T cell proliferation (31). Hyperthyroidism recovery was faster with appropriate Se supplementation in combination with thyrotacazole treatment than with thyrotacazole alone (32).

Some observational studies have shown a beneficial role of selenide in autoimmune diseases of the thyroid and other endocrine glands. This research may contribute to further understanding the role of Se status in inflammation, allergic reactions, and asthma (21). Se may influence the progression of autoimmune thyroid diseases by affecting immune responses (33). The pathogenesis of thyroid autoimmune diseases under low Se conditions is unclear. Possible mechanisms include a reduced cellular immune response, reduced production of interferon γ and other cytokines in Se deficiency, or an overreaction of the immune system. The balance between oxidation and antioxidant is an important feature of thyroid autoimmunity (34). Se may suppress the overreacting immune system activity or impair T lymphocytes immune function through antioxidant mechanisms involved in the pathogenesis of thyroid autoimmune diseases (22, 35, 36). In a series of animal studies, Se supplementation reduced thyroiditis prevalence and lymphocyte infiltration in the thyroid, affected the differentiation of T-cells, and upregulated regulatory T cells. A low-Se diet can enhance the development of autoantibodies against thyroglobulin (Tg) and thyroid peroxidase (TPO) (37, 38).

Se supplementation can suppress the Th1-dependent immune response, inhibiting the inflammatory response and destructive injury to thyroid. Se supplementation reportedly reduced serum thyroid peroxidase antibody (TPO-Ab) levels in patients with autoimmune thyroiditis treated with LT4 at 3 months, 6 months, and 12 months, as well as in untreated patients at 3 months. Not all studies are consistent, and different results may be due to regional differences in Se and iodine intake or to differences in inflammation severity and duration, study size, sample size, intervention duration, and the presence of other micronutrients. Therefore, the relationship between Se levels and autoimmune disease must be confirmed by large-scale, prospective trials (39).

The relationship between Se an cancer has been studied in a long time. Se supplementation may reduce the incidence of liver, esophagus, pancreas, prostate, colon and breast cancers (40, 41). Many data support the hypothesis that low levels of Se are associated with an increased incidence of thyroid cancer, especially PTC (42, 43). Se has a high concentration in thyroid tissue and is used to synthesize selenoproteins. Selenoprotein DIO catalyzes the conversion of T4 and T3, maintains steady-state levels of T3. Through cooperating with selenoprotein GPX and TXNRD, DIO also has antioxidant effects and protects thyroid gland against ROS (44). Se can also play its anticancer role by inducing death and apoptosis of cancer cells, producing superoxide radicals and triggering mitochondrial apoptosis. The anticancer effect of Se can selectively induce apoptosis of cancer cells without causing significant damage to normal cells (45). Therefore, the fluctuation of Se levels can affect the normal physiological process of thyroid gland and promote the development of pathological processes, including cancer. While this relationship has been clinically confirmed, the specific mechanism has not been clearly clarified (44, 46). Many animal model studies and epidemiological and interventional investigations have also shown that serum Se concentrations are related to the occurrence, development, and even metastasis of different types of cancer. Study limitations included access to retrospective data, a relatively small patient population, a short observation period, and the use of a single Se concentration level measurement.

Iodine and Se interact in a variety of ways in thyroid metabolism. Animal and human data have shown that Se concentration and selenoprotein expression are related to iodine intake (47). High iodine intake or exposure can reduce Se concentration and selenoprotein expression in the thyroid, while low iodine intake may be associated with increased levels of thyroid Se, selenoprotein, and blood Se markers (25, 26, 48, 49). Excessive Se intake will aggravate the consequences of iodine deficiency, while an appropriate Se supply can reduce the adverse effects of excess iodine on the thyroid and prevent thyroid inflammation, fibrosis, and destruction (21). The mechanism of thyroid damage in areas with combined iodine and Se deficiency is described as follows. GPX protects the thyroid from excessive H2O2 and plays an important role in the thyroid’s antioxidant defense. Iodine deficiency may stimulate H2O2 production, and Se deficiency may cause GPX activity to be insufficient to remove excess H2O2.

Se was first recognized as essential to health in 1957 (33). Maintaining physiological Se equilibrium through a reasonable diet or Se supplementation is required to prevent thyroid disease and to maintain overall health (22). The relationship between severe Se deficiency and thyroid dysfunction was only formally proposed in the 1990s, with an understanding of the link between Se and thyroid metabolism and the suggestion that Se supplementation might be useful in treating autoimmune thyroid diseases (50). Some studies have found that the increase of serum thyroglobulin antibody(TgAb) in patients with PTC after surgery is related to tumor recurrence and metastasis (51). Meanwhile, other studies have found that TgAb levels in patients with thyroid carcinoma after surgery can be controlled by oral Se yeast tablets (52). This can help avoid the missed diagnosis of patients with tumor recurrence or metastasis accompanied by low levels of Tg (caused by elevated TgAb). However, Se supplementation should consider the relatively narrow Se therapeutic dose range and the risks associated with acute and chronic overdose, especially in patients with type 2 diabetes (53). Adequate iodine levels should be established before increasing Se intake (20, 54).

### Iron

Iron (Ir) is essential to human health, as it participates in oxidation-reduction reactions and plays a role in oxygen transport in the body. The two initial steps in thyroid hormone synthesis are catalyzed by TPO, a heme-dependent protein. Ir deficiency adversely affects cognitive development, immune function, and pregnancy (55). Severe Ir deficiency can reduce TPO activity and interfere with thyroid hormone synthesis (56). A large number of animal and human studies have found that, with or without anemia, nutritional Ir deficiency can affect thyroid metabolism, reduce plasma total T4 and T3 levels, reduce peripheral T4-T3 conversion, and increase TSH levels (57). A survey found that 23-25% of school-aged children suffered from both goiter and Ir deficiency anemia (58).

Studies indicate that thyroid dysfunction, including hypothyroidism and hyperthyroidism, is associated with hemoglobin levels. Hyperthyroidism is associated with Ir deficiency anemia by altering Ir metabolism and utilization, increasing oxidative stress, and increasing hemolysis, thereby reducing the RBC survival rate (59, 60). One explanation for the co-existence of hypothyroidism and Ir deficiency anemia is that thyroid abnormalities and anemia share a common cause. Chronic inflammatory disease, malnutrition, and malabsorption can all lead to hypothyroidism, which is an adaptive response to energy deficiency. In addition, poor nutrition and malabsorption can lead to deficiencies in micronutrients such as Ir, vitamin B12, and folic acid, which are critical for red blood cell production, and iodine, which is essential for normal thyroid function.

Ir deficiency can also reduce TPO activity, which is the most common cause of thyroid dysfunction and anemia (61). Patients with hypothyroidism may experience Ir malabsorption. One study found that in hypothyroidism patients with low hemoglobin and low serum Ir levels, hemoglobin concentration increased after T4 replacement therapy. This increase was more significant when T4 was supplemented with Ir, possibly due to acidosis. Ir deficiency anemia may lead to changes in central nervous system, which may control the thyroid axis and may influence thyroid hormone level (62, 63). The link between hypothyroidism and anemia may be partly biological. The reason is that hypothyroidism reduces the need for oxygen transport and delivery to peripheral tissues (57). In conclusion, the mechanism of Ir deficiency’s impact on thyroid metabolism is not fully understood and requires further study.

There are many studies on Ir concentration and autoimmune diseases in pregnant and non-pregnant women. The prevalence of isolated positive TPOAb in non-pregnant women with iron-deficient is increasing. The more severe the Ir deficiency, the higher the prevalence of isolated positive TPOAb in pregnant women. However, Ir deficiency is not associated with positive TgAb alone (64, 65), and the mechanism remains unclear. Previous studies have shown that excess Ir can regulate and exaggerate autoimmune processes, which can induce reactive oxygen species production, oxidative stress, and lipid peroxidation, leading to demyelination of certain autoimmune diseases, such as autoimmune encephalomyelitis and multiple sclerosis (66–68). However, one study found that excess Ir was not associated with either positive TPOAb or TgAb, and further prospective studies are needed for verification (65). Patients with Hashimoto’s thyroiditis and subclinical hypothyroidism had lower serum Ir concentration and higher prevalence of Ir deficiency than healthy controls (69).

Ir homeostasis is essential to the biological processes of normal cells. The disruption of Ir homeostasis can lead to a variety of cell disorders, such as growth arrest. Excess Ir can damage proteins, DNA, and other cellular components (70, 71). Research on Ir and thyroid cancer is ongoing. Thyroid cancer cells secrete hepcidin, which can lead to decreased expression of Ferroportin (FPN) and increased intracellular Ir retention, thus promoting cancer proliferation. Most research suggests that there is a relationship between Ir and thyroid cancer, but the specific mechanism is unclear (72, 73).

In school-age children with Ir deficiency, thyroid volume was significantly reduced when Ir was supplemented with iodine or included in a food nutrition program (58, 74). The high prevalence of Ir deficiency in children in endemic goiter areas may reduce the effectiveness of iodized salt programs (57). Therefore, prevention of Ir deficiency anemia reduces Ir-related diseases and improves responses to iodized salt. Therefore, as for pregnant women and young children, further research on the advantages of Ir and iodine simultaneous supplementation is needed. New methods to further improve the stability and bioavailability of Ir and iodine in double-fortified salt are needed.

### Lithium

Iodine is concentrated in the thyroid gland through sodium iodide transporters. However, lithium’s mechanism of action on the thyroid gland is unclear. It has been reported that human administration of lithium (Li) can alter thyroid iodine uptake. This may be because Li competes with iodine transportation, which leads to a lower thyroid iodine uptake rate. Li can also affect iodine kinetics, causing iodine retention, induce hypothyroidism, and increase TSH secretion. Li has many effects on cell physiology. Its main effect on thyroid function is to induce hypothyroidism and goiter by inhibiting the release of thyroid hormone (75). Thyroid volume and goiter enlargement have been reported in patients treated with long-term lithium compared to controls (76). It also affects deiodination and iodine uptake (77).

Li effectively inhibits thyroid hormone release. It was first used in 1976 as an adjunct therapy for hyperthyroidism treated with radioactive iodine (78). Since then, more research has found that Li inhibits the release of thyroid hormones in cases of normal thyroid activity and hyperthyroidism (79). It can be used in the adjuvant treatment of I-131 in hyperthyroid patients, increasing the retention of radioactive iodine, effectively reducing the medication activity of hyperthyroidism, and reducing the increase in thyroid hormone concentration observed after treatment with radioactive iodine. It can be used in combination with thiamides for the adjuvant treatment of severe hyperthyroidism.

Although a study demonstrates that Li can be used as an adjuvant treatment for hyperthyroidism, some surveys have shown that the incidence of lithium-related asymptomatic thyroiditis and thyrotoxicosis is much higher than that of the general population (80). An epidemiological study of Li concluded that long-term Li intake was associated with an increase in thyrotoxicosis (80). This may be because Li therapy reduces thyroid hormones and covers the signs of hyperthyroidism, which occurs when the body adapts to Li therapy. Li can also directly damage thyroid cells and release Tg and thyroid hormones into circulation, making the thyroid temporarily hyperactive.

Li is reportedly an adjuvant element in the radioactive iodine treatment of metastatic well-differentiated thyroid cancer and the ablation of postoperative residual tissue for low-risk thyroid cancer (81–83). However, at present, there is no evidence that Li has any beneficial effect on the control of the development of differentiated thyroid cancer. Some studies have found that Li has not a significant effect as an adjuvant drug (84).

It was first proposed that patients treated with Li would develop a goiter, followed by hypothyroidism (85). However, the prevalence of Li-related hypothyroidism varies depending on the study population, laboratory evaluations, and environmental factors. Common clinical side effects of Li related drug are reported in up to 40% of goiter cases and 20% of hypothyroid cases. The incidence of complications varies greatly based on the patient and analysis methods (80). Li inhibits thyroid hormone production, leading to elevated TSH levels and increased risk of goiter. Wnt/β-catenin signaling may play an important role in Li-associated goiters (86). Li promotes β-catenin-mediated thyroid cell proliferation (87) and can accelerate the progression of existing thyroiditis, as evidenced by an increase in circulating antibodies. On the contrary, some studies suggest there is no increase in the prevalence of thyroiditis or thyroid antibody levels in patients treated with Li (88, 89).

Li-related hypothyroidism may be associated with autoimmunity, which may be due to inhibition of thyroid hormone secretion by Li. Thyroid function should be monitored long-term in the course of Li treatment. Some patients experience subclinical hypothyroidism, which may develop within weeks, months, or years of starting Li and may include atypical features such as myxoedema (90). Meanwhile, some studies indicate that iodine and Li have a synergistic effect on hypothyroidism (91, 92). Dietary iodine concentration, the body’s innate iodine levels, thyroid hormones, and their interactions with chronic Li therapy can all influence hypothyroidism.

### Copper

As an oxidation-reduction active element, copper (Co) maintains thyroid activity and lipid metabolism. Co prevents T4 overabsorption and controls calcium levels. The relationship between Co and thyroid function has been described in the literature. Animal studies indicate that relatively high levels of Co are associated with hypothyroidism while relatively low levels are associated with hyperthyroidism (28, 93). With increased serum Co levels, TSH level decreases monotonically (24). Some studies have found that Co is positively correlated with thyroid hormone and can stimulate thyroid hormone production (93). Co reduction may increase oxidative stress in thyroid cells, resulting in reduced thyroid hormone synthesis and decreased circulating thyroid hormone levels. Conversely, thyroid hormones can also affect blood Co concentration. Experimental studies in mice suggest that thyroid hormones can regulate blood Co levels by increasing Co output from the liver. T3 treatment revises the expression of ceruloplasmin in mice, leading to an increase in serum Co levels (94). A human study showed that radioactive iodine treatment in patients with hyperthyroidism reduced thyroid hormone levels and, subsequently, serum Co levels (95).

Very little is known about the relationships between Co and thyroid autoimmune diseases. It has been reported that a high serum Co concentration is positively correlated with the presence of thyroid autoantibodies (96). In another study, Co levels have not been associated with thyroid autoimmune inflammation and thyroid autoantibodies (97).

Co is thought to start angiogenesis in tumor cells (98). High concentrations of Co can induce growth, proliferation, and cancer through DNA damage by toxic hydroxyl radicals (99). The Co concentration of healthy thyroid tissue is significantly higher than that in the benign thyroid tissue (100, 101), and some studies indicate that the serum Co level in patients with benign thyroid disease after surgery is significantly lower than before surgery. Co is associated with thyroid cancer, and inhibition of MEK1/2 kinase activity by Co chelating agents may be used in combination with other MAPK pathway inhibitors to treat BRAF mutation-positive cancers and cancers resistant to BRAFV600E and MEK1/2 inhibitors (102).

### Zinc

Zinc (Zn) is essential for human health and plays a role in gene expression, cell division and growth, and in a variety of enzymes involved in immune and reproductive functioning. Zn deficiency can affect the physical development of children and increase the risk of several infections (103). There have been many studies on Zn and thyroid hormone levels, and both hypothyroidism and hyperthyroidism are reportedly associated with low Zn concentrations (104). Low Zn levels are believed to be associated with hypothyroidism and high Zn levels with hyperthyroidism (105). One study found a significant positive correlation between thyroid autoantibodies and Zn in thyroid autoimmune patients (106). Zn can also affect thyroid volume. The volume of thyroid was positively correlated with Zn concentration (74, 107).

Serum Zn concentrations are significantly reduced in many malignant tumors (108), including thyroid cancer. Zn levels in PTC and follicular carcinoma are lower than that in healthy individuals (108–110). Changes in trace elements in the serum and thyroid tissues may be related to thyroid cancer pathogenesis. Zn is essential for thyroid hormone metabolism and has a potential relationship with cancer. Therefore, it is critical to assess micronutrient deficiencies to optimize targeted nutritional therapy for thyroid cancer patients.

Dietary Zn supplementation, initially used to restore immune function, also improves thyroid function, such as reduced TSH levels. Compared with the single application of Li, the application of Li and Zn generates higher levels of T3 and T4, suggesting that Zn has a regulatory effect on thyroid hormones. Therefore, Zn has some potential protective effect to alleviate thyroid function changes after Li supplementation (111).

### Manganese

The trace element manganese (Mn) is a co-factor of many enzymes and has a variety of functions. Mn may interfere with the binding, transport, and activity of thyroid hormones at the tissue level (112, 113). Mn deficiency is rare in humans as we generally maintain a stable tissue level of this trace element (114). The effect of Mn on the thyroid is not well understood. A study clarified that serum Mn levels are strongly correlated with thyroid hormones, as a high Mn concentration reduces free T3 and free T4 levels, causing hypothyroidism (114, 115). Mn may affect the thyroid hormone levels by regulating deiodinases, which converts T4 to T3. The thyroids of female mice treated with excessive amounts of Mn were enlarged (116). Children with nontoxic goiter has a higher concentration of serum manganese (117). The concentration of manganese in the serum and thyroid tissues of patients with Hashimoto’s thyroiditis is higher than in those with normal thyroid (118, 119)

Mn deficiency has an impact not only on thyroid hormone metabolism, but also on other physiological processes such as nervous system development (120). Dopamine is a regulator of TSH secretion, and Mn destroys dopaminergic neurons by influencing TSH and thyroid hormones, eventually leading to neurodevelopmental defects (112). The increased incidence of thyroid cancer has been associated with a number of micronutrients, including Mn (121). The concentration of Mn in thyroid tissue is increased in patients with thyroid cancer compared with those with benign thyroid (122). The mutagenicity and carcinogenicity of Mn may be related to oxidative stress (123).

### Magnesium

Magnesium (Mg) plays a central role in thyroid disease. Mg is related to the stabilization of the structure of nucleic acids and seems also involved in DNA replication, transcription, and repair. Therefore, any Mg deficiency can lead to the development of tumors through DNA mutations (124). Serum Mg levels are closely related to thyroid cancer, and malignant tumors usually have higher Mg levels than normal tissues (125, 126). Meanwhile, serum Mg levels in thyroid cancer patients are lower than in healthy people (127). Mg may influence the development of cancer through its association with inflammation and/or free radicals, which may lead to DNA oxidative damage and cancer formation. Mg-deficient animals show a higher sensitivity to oxidative stress *in vivo* and their tissues are more susceptible to peroxides *in vitro (* 128). Also, experimental data indicate that high doses of Mg can increase thyroid activity (129), an Mg deficiency affects the bioavailability and tissue distribution of Se, resulting in decreased Se levels (130).

Dietary Mg deficiency can affect thyroid activity. Thyroid volume increased and total T4 level decreased in rats with Mg deficiency, with no significant changes in T3. Mg is essential for thyroid utilization of iodine and the conversion of inactive T4 into active T3 (131). These data support Mg’s association with thyroid function.

### Cadmium

Cadmium (Cd) is listed as one of 126 priority pollutants and a kind of carcinogen. There are insufficient studies on its effects on the thyroid, but it has been reported that chronic Cd poisoning is common in cases of colloid cystic goiter, adenomatous follicular hyperplasia with low dysplasia and reduced Tg secretion, and diffuse nodular hyperplasia and hypertrophy of parafollicular cells (132–134).

Chronic Cd exposure reportedly affects thyroid structure and function, damaging follicular cells and parafollicular cells, and Cd accumulation in the thyroid gland is associated with abnormal thyroid hormone levels and thyroid lesions. Higher levels of Cd exposure can increase TSH, possibly because Cd regulates the production and secretion of thyroid hormones, which can decrease T4 levels. Cd affects thyroid hormone metabolism, including the central and peripheral nervous system.

Cd exposure causes changes in circulating thyroid hormone or TSH levels due to the hypothalamic-pituitary-thyroid axis or the interruption of thyroid hormone transport and peripheral metabolic inactivation (135, 136). Cd may increase the risk of goiter at certain concentrations, which can affect the volume of thyroid gland (121). Blood Cd levels were positively correlated with female TgAb (137). Cd and its compounds are recognized as carcinogens, although the role of Cd as a human thyroid carcinogen remains unclear, research indicates that the Cd in thyroid tissues of patients with advanced thyroid cancer is higher. The accumulation of Cd in thyroid tissues may lead to the progression and aggravation of thyroid cancer in Korean women (138–140).

### Molybdenum

Many studies have found that molybdenum (Mo) is related to thyroid metabolism. In males, Mo level is positively correlated with the TSH level. Mo can interact with thyroid hormone receptors to affect thyroid hormone levels (141), and Mo treatment can cause histological changes to the thyroid follicular cells in rats (139), which suggests that Mo can alter thyroid hormone levels. There are few studies on Mo and thyroid volume. Iodine deficiency may be accompanied by Mo deficiency, and some patients with endemic goiter should be supplemented with trace elements including Mo on the basis of iodine supportive therapy (142). An increase in dietary Mo may accelerate or promote cell transformation, thus acting as a tumor promoter, but not a carcinogen (141). Chronic exposure to certain levels of boron, Cd and Mo in rats with hypothyroidism has been accelerated malignant transformation of thyroid cells. There may be some association between Mo and thyroid cancer (137).

## Other trace elements

The incidence of thyroid disease has dramatically increased over the last few decades. Although the etiology of thyroid disease is not fully understood, the role of trace element deficiency or excess is being recognized. In addition to the above mentioned, the role of human thyroid metabolism and function also requires other trace elements, such as arsenic (As), plumbum (Pb), and mercury (Hg). The As and Pb concentration in the thyroid tissue of Hashimoto’s thyroiditis patients was significantly increased (143), and the concentration of As and Pb in maternal urine was negatively correlated with FT3 and FT3/FT4 levels in the mother. This concentration also affects neonatal thyroid hormone levels (144, 145). It has also been suggested that higher levels of lead exposure may be positively correlated with total T3 (both male and female) and total T4 (female) concentrations (146). A higher level of Pb in the blood of teenagers with nontoxic goiter in comparison with healthy adolescents was found in a study (117).

Although epidemiological investigations indicate that As is a human carcinogen, its mechanism of action (147) and the relationship between As and thyroid cancer is not fully understood. As can inhibit thyroid homeostasis directly and alter the expression of related genes (148). Furthermore, it has been speculated that As disrupts Se’s anticancer activity (122). As The accumulation of Pb in the thyroid gland leads to the destruction of the follicular cell structure and thyroid dysfunction (149). Hg is associated with a decrease in T3 and T4 levels, while some surveys show no correlation with TSH levels. However, serum TSH concentration may be higher in people exposed to Hg (150, 151). Hg exposure is associated with positive cell autoimmunity and is positively correlated with TgAb and TPOAb (152). Hg may also be a potential thyroid carcinogen. Long-term follow-up of thyroid conditions in exposed populations is recommended to further study the effects of occupational Hg exposure on the thyroid.

## Conclusion

Various trace elements are important to thyroid metabolism and function, and correlate with thyroid autoimmunity and tumors (**Table 1**). For example, there is strong evidence that Ir and iodine are closely related to thyroid metabolism and that serum Se, Zn, and Co impact thyroid hormone levels. Evidence also suggests that Zn, Co and Cd may interact with one another, trace element abnormalities may impair thyroid iodine uptake. Preventing trace element deficiency may not only reduce the diseases associated with that trace elements but also improve the effectiveness of other trace elements. The prevalence of trace elements deficiencies may reduce the effectiveness of ongoing public health programs due to trace element interactions. Meanwhile, further research is warranted for thyroid-related trace elements, which in order to provide better clinical diagnosis and treatment strategies of thyroid disease in future.

**Table 1:** Effect of trace elements on thyroid diseases.

Trace elements	Thyroid volume	Thyroid function	Autoimmune thyroiditis	Thyroid carcinoma	Types of evidence	Corresponding references
I High intake Low intake	- +	↑/↓ ↑/↓	+ -	+ +/-	1,2,3	(3–6, 9, 10, 13–18)
Se High intake Low intake	- +	↑ ↓	- +	- +	1,2,3	(20, 22, 26, 27, 35–38, 42, 43)
Ir High intake Low intake	+ -	↑/↓ ↓	+ +	+ -	1,2,3	(59, 60, 62–65, 72–74)
Li High intake Low intake	+ -	↓ ↑/↓	+ -	- +	1,2,3	(75, 76, 79–83, 85, 88–92)
Co High intake Low intake	+ -	↑/↓ ↓/↑	+ -	+ -	1,2	(93–97, 99, 102)
Zn High intake Low intake	+ -	↑/↓ ↓/↑	+ -	- +	1,2	(74, 104–111)
Mn High intake Low intake	+ -	↑ ↓	+ -	+ -	1,2	(114–119, 121–123)
Mg High intake Low intake	+ -	↑ ↓	—— ——	+ +	1,2	(125–131)
Cd High intake Low intake	+ -	↓ ↑	+ -	+ -	1,2	(121, 132–140)
Mo High intake Low intake	- +	↓ ↑	—— ——	+ -	1,2	(137, 139, 141, 142)
As High intake Low intake	—— ——	↓ ↑	+ -	—— ——	1	(122, 143–145, 147, 148)
Pb High intake Low intake	+ -	↓/↑ ↑/↓	+ -	+ -	1	(117, 143–146, 149)
Hg High intake Low intake	—— ——	↓ ↑	+ -	+ -	1,2	(150–152)

↑ : Hyperthyroidism, ↓ : Hypothyroidism, + : Promotion, - : Inhibition, —— : Not Clear, Type of evidence: 1: population studies, 2: animal studies, 3: intervention trial.

## Author contributions

All authors contributed to the article and approved the submitted version.

## Conflict of interest

The authors declare that the research was conducted in the absence of any commercial or financial relationships that could be construed as a potential conflict of interest.

## Publisher’s note

All claims expressed in this article are solely those of the authors and do not necessarily represent those of their affiliated organizations, or those of the publisher, the editors and the reviewers. Any product that may be evaluated in this article, or claim that may be made by its manufacturer, is not guaranteed or endorsed by the publisher.

## References

[1] Zaichick V Tsyb AF Vtyurin BM. Trace elements and thyroid cancer. Analyst (1995) 120(3):817–21. doi: 10.1039/an9952000817 7741233

[2] Cooper DS. Subclinical thyroid disease: consensus or conundrum? Clin Endocrinol (Oxf) (2004) 60(4):410–2. doi: 10.1111/j.1365-2265.2004.02031.x 15049953

[3] Zimmermann MB Boelaert K. Iodine deficiency and thyroid disorders. Lancet Diabetes Endocrinol (2015) 3(4):286–95. doi: 10.1016/S2213-8587(14)70225-6 25591468

[4] Laurberg P Cerqueira C Ovesen L Rasmussen LB Perrild H Andersen S. Iodine intake as a determinant of thyroid disorders in populations. Best Pract Res Clin Endocrinol Metab (2010) 24(1):13–27. doi: 10.1016/j.beem.2009.08.013 20172467

[5] Liu XH Chen GG Vlantis AC van Hasselt CA. Iodine mediated mechanisms and thyroid carcinoma. Crit Rev Clin Lab Sci (2009) 46(5-6):302–18. doi: 10.3109/10408360903306384 19958216

[6] Petersen M Bulow Pedersen I Knudsen N Andersen S Jorgensen T Perrild H. Changes in subtypes of overt thyrotoxicosis and hypothyroidism following iodine fortification. Clin Endocrinol (Oxf) (2019) 91(5):652–9. doi: 10.1111/cen.14072 31400012

[7] Vagenakis AG Braverman LE. Adverse effects of iodides on thyroid function. Med Clin North Am (1975) 59(5):1075–88. doi: 10.1016/S0025-7125(16)31958-7 1099355

[8] Farebrother J Zimmermann MB Andersson M. Excess iodine intake: sources, assessment, and effects on thyroid function. Ann N Y Acad Sci (2019) 1446(1):44–65. doi: 10.1111/nyas.14041 30891786

[9] Aghini Lombardi F Fiore E Tonacchera M Antonangeli L Rago T Frigeri M. The effect of voluntary iodine prophylaxis in a small rural community: the pescopagano survey 15 years later. J Clin Endocrinol Metab (2013) 98(3):1031–9. doi: 10.1210/jc.2012-2960 23436921

[10] Teng W Shan Z Teng X Guan H Li Y Teng D. Effect of iodine intake on thyroid diseases in China. N Engl J Med (2006) 354(26):2783–93. doi: 10.1056/NEJMoa054022 16807415

[11] Duntas LH. The catalytic role of iodine excess in loss of homeostasis in autoimmune thyroiditis. Curr Opin Endocrinol Diabetes Obes (2018) 25(5):347–52. doi: 10.1097/MED.0000000000000425 30124478

[12] Pedersen IB Knudsen N Carle A Vejbjerg P Jorgensen T Perrild H. A cautious iodization programme bringing iodine intake to a low recommended level is associated with an increase in the prevalence of thyroid autoantibodies in the population. Clin Endocrinol (Oxf) (2011) 75(1):120–6. doi: 10.1111/j.1365-2265.2011.04008.x 21521277

[13] Ruggeri RM Trimarchi F. Iodine nutrition optimization: are there risks for thyroid autoimmunity? J Endocrinol Invest (2021) 44(9):1827–35. doi: 10.1007/s40618-021-01548-x 33683664

[14] Wang F Wang Y Wang L Wang X Sun C Xing M. Strong association of high urinary iodine with thyroid nodule and papillary thyroid cancer. Tumour Biol (2014) 35(11):11375–9. doi: 10.1007/s13277-014-2397-8 25119588

[15] Guan H Ji M Bao R Yu H Wang Y Hou P. Association of high iodine intake with the T1799A BRAF mutation in papillary thyroid cancer. J Clin Endocrinol Metab (2009) 94(5):1612–7. doi: 10.1210/jc.2008-2390 19190105

[16] Boltze C Brabant G Dralle H Gerlach R Roessner A Hoang-Vu C. Radiation-induced thyroid carcinogenesis as a function of time and dietary iodine supply: An *in vivo* model of tumorigenesis in the rat. Endocrinology (2002) 143(7):2584–92. doi: 10.1210/endo.143.7.8914 12072390

[17] Kim HJ Kim NK Park HK Byun DW Suh K Yoo MH. Strong association of relatively low and extremely excessive iodine intakes with thyroid cancer in an iodine-replete area. Eur J Nutr (2017) 56(3):965–71. doi: 10.1007/s00394-015-1144-2 26746218

[18] Burgess JR Dwyer T McArdle K Tucker P Shugg D. The changing incidence and spectrum of thyroid carcinoma in Tasmania (1978-1998) during a transition from iodine sufficiency to iodine deficiency. J Clin Endocrinol Metab (2000) 85(4):1513–7. doi: 10.1210/jcem.85.4.6554 10770190

[19] Wang B He W Li Q Jia X Yao Q Song R. U-Shaped relationship between iodine status and thyroid autoimmunity risk in adults. Eur J Endocrinol (2019) 181(3):255–66. doi: 10.1530/EJE-19-0212 31252413

[20] Liu Y Huang H Zeng J Sun C. Thyroid volume, goiter prevalence, and selenium levels in an iodine-sufficient area: A cross-sectional study. BMC Public Health (2013) 13:1153. doi: 10.1186/1471-2458-13-1153 24321191 PMC3878896

[21] Kohrle J. Selenium and the thyroid. Curr Opin Endocrinol Diabetes Obes (2015) 22(5):392–401. doi: 10.1097/MED.0000000000000190 26313901

[22] Duntas LH Benvenga S. Selenium: an element for life. Endocrine (2015) 48(3):756–75. doi: 10.1007/s12020-014-0477-6 25519493

[23] Gupta S Jaworska-Bieniek K Lubinski J Jakubowska A. Can selenium be a modifier of cancer risk in CHEK2 mutation carriers? Mutagenesis (2013) 28(6):625–9. doi: 10.1093/mutage/get050 24106007

[24] Jain RB. Thyroid function and serum copper, selenium, and zinc in general U.S. population. Biol Trace Elem Res (2014) 159(1-3):87–98. doi: 10.1007/s12011-014-9992-9 24789479

[25] Bulow Pedersen I Knudsen N Carle A Schomburg L Kohrle J Jorgensen T. Serum selenium is low in newly diagnosed graves’ disease: a population-based study. Clin Endocrinol (Oxf) (2013) 79(4):584–90. doi: 10.1111/cen.12185 23448365

[26] Kohrle J. Selenium and the thyroid. Curr Opin Endocrinol Diabetes Obes (2013) 20(5):441–8. doi: 10.1097/01.med.0000433066.24541.88 23974773

[27] Derumeaux H Valeix P Castetbon K Bensimon M Boutron-Ruault MC Arnaud J. Association of selenium with thyroid volume and echostructure in 35- to 60-year-old French adults. Eur J Endocrinol (2003) 148(3):309–15. doi: 10.1530/eje.0.1480309 12611611

[28] Wertenbruch T Willenberg HS Sagert C Nguyen TB Bahlo M Feldkamp J. Serum selenium levels in patients with remission and relapse of graves’ disease. Med Chem (2007) 3(3):281–4. doi: 10.2174/157340607780620662 17504200

[29] Khong JJ Goldstein RF Sanders KM Schneider H Pope J Burdon KP. Serum selenium status in graves’ disease with and without orbitopathy: A case-control study. Clin Endocrinol (Oxf) (2014) 80(6):905–10. doi: 10.1111/cen.12392 24372054

[30] Dehina N Hofmann PJ Behrends T Eckstein A Schomburg L. Lack of association between selenium status and disease severity and activity in patients with graves’ ophthalmopathy. Eur Thyroid J (2016) 5(1):57–64. doi: 10.1159/000442440 27099840 PMC4836115

[31] Wang Y Zhao F Rijntjes E Wu L Wu Q Sui J. Role of selenium intake for risk and development of hyperthyroidism. J Clin Endocrinol Metab (2019) 104(2):568–80. doi: 10.1210/jc.2018-01713 30265356

[32] Wang L Wang B Chen SR Hou X Wang XF Zhao SH. Effect of selenium supplementation on recurrent hyperthyroidism caused by graves’ disease: A prospective pilot study. Horm Metab Res (2016) 48(9):559–64. doi: 10.1055/s-0042-110491 27392116

[33] Winther KH Rayman MP Bonnema SJ Hegedus L. Selenium in thyroid disorders - essential knowledge for clinicians. Nat Rev Endocrinol (2020) 16(3):165–76. doi: 10.1038/s41574-019-0311-6 32001830

[34] Ruggeri RM CampennÌ A Giuffrida G Casciaro M Barbalace MC Hrelia S. Oxidative stress as a key feature of autoimmune thyroiditis: An update. Minerva Endocrinol (2020) 45(4):326–44. doi: 10.23736/S0391-1977.20.03268-X 32969631

[35] Stoedter M Renko K Hog A Schomburg L. Selenium controls the sex-specific immune response and selenoprotein expression during the acute-phase response in mice. Biochem J (2010) 429(1):43–51. doi: 10.1042/BJ20091868 20370716

[36] Broome CS McArdle F Kyle JA Andrews F Lowe NM Hart CA. An increase in selenium intake improves immune function and poliovirus handling in adults with marginal selenium status. Am J Clin Nutr (2004) 80(1):154–62. doi: 10.1093/ajcn/80.1.154 15213043

[37] Wang W Xue H Li Y Hou X Fan C Wang H. Effects of selenium supplementation on spontaneous autoimmune thyroiditis in NOD.H-2h4 mice. Thyroid (2015) 25(10):1137–44. doi: 10.1089/thy.2014.0568 26121912

[38] McLachlan SM Aliesky H Banuelos B Hee SSQ Rapoport B. Variable effects of dietary selenium in mice that spontaneously develop a spectrum of thyroid autoantibodies. Endocrinology (2017) 158(11):3754–64. doi: 10.1210/en.2017-00275 PMC5695827 28938453

[39] Wichman J Winther KH Bonnema SJ Hegedus L. Selenium supplementation significantly reduces thyroid autoantibody levels in patients with chronic autoimmune thyroiditis: A systematic review and meta-analysis. Thyroid (2016) 26(12):1681–92. doi: 10.1089/thy.2016.0256 27702392

[40] Davis CD Tsuji PA Milner JA. Selenoproteins and cancer prevention. Annu Rev Nutr (2012) 32:73–95. doi: 10.1146/annurev-nutr-071811-150740 22404120

[41] Renko K Hofmann PJ Stoedter M Hollenbach B Behrends T Kohrle J. Down-regulation of the hepatic selenoprotein biosynthesis machinery impairs selenium metabolism during the acute phase response in mice. FASEB J (2009) 23(6):1758–65. doi: 10.1096/fj.08-119370 19136613

[42] Glattre E Nygard JF Aaseth J. Selenium and cancer prevention: observations and complexity. J Trace Elem Med Biol (2012) 26(2-3):168–9. doi: 10.1016/j.jtemb.2012.04.021 22664335

[43] Combs GF Jr. Current evidence and research needs to support a health claim for selenium and cancer prevention. J Nutr (2005) 135(2):343–7. doi: 10.1093/jn/135.2.343 15671240

[44] de Oliveira Maia M Batista BAM Sousa MP de Souza LM Maia CSC. Selenium and thyroid cancer: a systematic review. Nutr Cancer (2020) 72(8):1255–63. doi: 10.1080/01635581.2019.1679194 31635488

[45] Xiang N Zhao R Zhong W. Sodium selenite induces apoptosis by generation of superoxide via the mitochondrial-dependent pathway in human prostate cancer cells. Cancer Chemother Pharmacol (2009) 63(2):351–62. doi: 10.1007/s00280-008-0745-3 PMC2592502 18379781

[46] Rayman MP. Selenium and human health. Lancet (2012) 379(9822):1256–68. doi: 10.1016/S0140-6736(11)61452-9 22381456

[47] Kohrle J Jakob F Contempre B Dumont JE. Selenium, the thyroid, and the endocrine system. Endocr Rev (2005) 26(7):944–84. doi: 10.1210/er.2001-0034 16174820

[48] Brauer VF Schweizer U Kohrle J Paschke R. Selenium and goiter prevalence in borderline iodine sufficiency. Eur J Endocrinol (2006) 155(6):807–12. doi: 10.1530/eje.1.02302 17132749

[49] Drutel A Archambeaud F Caron P. Selenium and the thyroid gland: more good news for clinicians. Clin Endocrinol (Oxf) (2013) 78(2):155–64. doi: 10.1111/cen.12066 23046013

[50] YB W ZY D SW X XY M. Clinical significance of serum thyroglobulin level in patients with differentiated thyroid carcinoma. J Clin Lab Med (2019) 34(5):133–134+138. doi: 10.3969/j.issn.1671-7414.2019.05.033

[51] Feng XL Qu YK Zhao MT Wang QY WD J. Effect of selenium yeast on TgAb and TG in patients with differentiated thyroid carcinoma after total resection medical diet and health. Med Food Ther Health (2021) 19(2):137–8.

[52] Stuss M Michalska-Kasiczak M Sewerynek E. The role of selenium in thyroid gland pathophysiology. Endokrynol Pol (2017) 68(4):440–65. doi: 10.5603/EP.2017.0051 28819948

[53] Contempre B Duale NL Dumont JE Ngo B Diplock AT Vanderpas J. Effect of selenium supplementation on thyroid hormone metabolism in an iodine and selenium deficient population. Clin Endocrinol (Oxf) (1992) 36(6):579–83. doi: 10.1111/j.1365-2265.1992.tb02268.x 1424183

[54] Hess SY. The impact of common micronutrient deficiencies on iodine and thyroid metabolism: the evidence from human studies. Best Pract Res Clin Endocrinol Metab (2010) 24(1):117–32. doi: 10.1016/j.beem.2009.08.012 20172476

[55] Hurrell RF. Bioavailability of iodine. Eur J Clin Nutr (1997) 51 Suppl 1:S9–12. 9023472

[56] Zimmermann MB. The influence of iron status on iodine utilization and thyroid function. Annu Rev Nutr (2006) 26:367–89. doi: 10.1146/annurev.nutr.26.061505.111236 16602928

[57] Zimmermann MB Zeder C Chaouki N Saad A Torresani T Hurrell RF. Dual fortification of salt with iodine and microencapsulated iron: a randomized, double-blind, controlled trial in Moroccan schoolchildren. Am J Clin Nutr (2003) 77(2):425–32. doi: 10.1093/ajcn/77.2.425 12540404

[58] Yucel R Ozdemir S Dariyerli N Toplan S Akyolcu MC Yigit G. Erythrocyte osmotic fragility and lipid peroxidation in experimental hyperthyroidism. Endocrine (2009) 36(3):498–502. doi: 10.1007/s12020-009-9251-6 19851894

[59] Asl SZ Brojeni NK Ghasemi A Faraji F Hedayati M Azizi F. Alterations in osmotic fragility of the red blood cells in hypo- and hyperthyroid patients. J Endocrinol Invest (2009) 32(1):28–32. doi: 10.1007/BF03345674 19337011

[60] Wopereis DM Du Puy RS van Heemst D Walsh JP Bremner A Bakker SJL. The relation between thyroid function and anemia: A pooled analysis of individual participant data. J Clin Endocrinol Metab (2018) 103(10):3658–67. doi: 10.1210/jc.2018-00481 PMC6179176 30113667

[61] Beard JL Brigham DE Kelley SK Green MH. Plasma thyroid hormone kinetics are altered in iron-deficient rats. J Nutr (1998) 128(8):1401–8. doi: 10.1093/jn/128.8.1401 9687562

[62] Smith SM Johnson PE Lukaski HC. In vitro hepatic thyroid hormone deiodination in iron-deficient rats: Effect of dietary fat. Life Sci (1993) 53(8):603–9. doi: 10.1016/0024-3205(93)90268-8 8350675

[63] Veltri F Decaillet S Kleynen P Grabczan L Belhomme J Rozenberg S. Prevalence of thyroid autoimmunity and dysfunction in women with iron deficiency during early pregnancy: is it altered? Eur J Endocrinol (2016) 175(3):191–9. doi: 10.1530/EJE-16-0288 27450694

[64] Zhang HY Teng XC Shan ZY Wang ZJ Li CY Yu XH. Association between iron deficiency and prevalence of thyroid autoimmunity in pregnant and non-pregnant women of childbearing age: a cross-sectional study. Chin Med J (Engl) (2019) 132(18):2143–9. doi: 10.1097/CM9.0000000000000409 PMC6797140 31478926

[65] Dabbaghmanesh MH Sadegholvaad A Ejtehadi F Ranjbar-Omrani G. The role of iron deficiency in persistent goiter. Arch Iran Med (2008) 11(2):157–61. doi: 108112/AIM.007 18298292

[66] Antel JP Moumdjian R. Paraneoplastic syndromes: a role for the immune system. J Neurol (1989) 236(1):1–3. doi: 10.1007/BF00314208 2644394

[67] Izawa T Yamate J Franklin RJ Kuwamura M. Abnormal iron accumulation is involved in the pathogenesis of the demyelinating dmy rat but not in the hypomyelinating mv rat. Brain Res (2010) 1349:105–14. doi: 10.1016/j.brainres.2010.06.030 20599839

[68] Erdal M Sahin M Hasimi A Uckaya G Kutlu M Saglam K. Trace element levels in hashimoto thyroiditis patients with subclinical hypothyroidism. Biol Trace Elem Res (2008) 123(1-3):1–7. doi: 10.1007/s12011-008-8117-8 18322655

[69] Muckenthaler MU Galy B Hentze MW. Systemic iron homeostasis and the iron-responsive element/iron-regulatory protein (IRE/IRP) regulatory network. Annu Rev Nutr (2008) 28:197–213. doi: 10.1146/annurev.nutr.28.061807.155521 18489257

[70] Anderson CP Shen M Eisenstein RS Leibold EA. Mammalian iron metabolism and its control by iron regulatory proteins. Biochim Biophys Acta (2012) 1823(9):1468–83. doi: 10.1016/j.bbamcr.2012.05.010 PMC3675657 22610083

[71] Zhou Q Chen J Feng J Wang J. E4BP4 promotes thyroid cancer proliferation by modulating iron homeostasis through repression of hepcidin. Cell Death Dis (2018) 9(10):987. doi: 10.1038/s41419-018-1001-3 30250199 PMC6155336

[72] Chisholm M. The association between webs, iron and post-cricoid carcinoma. Postgrad Med J (1974) 50(582):215–9. doi: 10.1136/pgmj.50.582.215 PMC2495558 4449772

[73] Lazarus JH. Lithium and thyroid. Best Pract Res Clin Endocrinol Metab (2009) 23(6):723–33. doi: 10.1016/j.beem.2009.06.002 19942149

[74] El-Fadeli S Bouhouch S Skalny AV Barkouch Y Pineau A Cherkaoui M. Effects of imbalance in trace element on thyroid gland from Moroccan children. Biol Trace Elem Res (2016) 170(2):288–93. doi: 10.1007/s12011-015-0485-2 26315305

[75] Ozsoy S Mavili E Aydin M Turan T Esel E. Ultrasonically determined thyroid volume and thyroid functions in lithium-naïve and lithium-treated patients with bipolar disorder: A cross-sectional and longitudinal study. Hum Psychopharmacol (2010) 25(2):174–8. doi: 10.1002/hup.1093 20196184

[76] Bauer M lumentritt H Finke R Schlattmann P Adli M Baethge C. Using ultrasonography to determine thyroid size and prevalence of goiter in lithium-treated patients with affective disorders. J Affect Disord (2007) 104(1-3):45–51. doi: 10.1016/j.jad.2007.01.033 17346802

[77] Turner JG Brownlie BE Rogers TG. Lithium as an adjunct to radioiodine therapy for thyrotoxicosis. Lancet (1976) 1(7960):614–5. doi: 10.1016/S0140-6736(76)90419-0 55895

[78] Vandendriessche B Lapauw B Kaufman JM Fiers T. A practical approach towards the evaluation of aberrant thyroid function tests. Acta Clin Belg (2020) 75(2):155–62. doi: 10.1080/17843286.2019.1577531 30806594

[79] Miller KK Daniels GH. Association between lithium use and thyrotoxicosis caused by silent thyroiditis. Clin Endocrinol (Oxf) (2001) 55(4):501–8. doi: 10.1046/j.1365-2265.2001.01381.x 11678833

[80] Liu YY van der Pluijm G Karperien M Stokkel MP Pereira AM Morreau J. Lithium as adjuvant to radioiodine therapy in differentiated thyroid carcinoma: clinical and in vitro studies. Clin Endocrinol (Oxf) (2006) 64(6):617–24. doi: 10.1111/j.1365-2265.2006.02515.x 16712662

[81] Luo H Tobey A Auh S Cochran C Zemskova M Reynolds J. The effect of lithium on the progression-free and overall survival in patients with metastatic differentiated thyroid cancer undergoing radioactive iodine therapy. Clin Endocrinol (Oxf) (2018) 89(4):481–8. doi: 10.1111/cen.13806 PMC6138537 29972703

[82] Yamazaki CA Padovani RP Biscolla RP Ikejiri ES Marchetti RR Castiglioni ML. Lithium as an adjuvant in the postoperative ablation of remnant tissue in low-risk thyroid carcinoma. Thyroid (2012) 22(10):1002–6. doi: 10.1089/thy.2011.0372 22953991

[83] Bal CS Kumar A Pandey RM. A randomized controlled trial to evaluate the adjuvant effect of lithium on radioiodine treatment of hyperthyroidism. Thyroid (2002) 12(5):399–405. doi: 10.1089/105072502760043486 12097201

[84] Schou M Amdisen A Eskjaer Jensen S Olsen T. Occurrence of goitre during lithium treatment. Br Med J (1968) 3(5620):710–3. doi: 10.1136/bmj.3.5620.710 PMC1989588 17016877

[85] Klein PS Melton DA. A molecular mechanism for the effect of lithium on development. Proc Natl Acad Sci U.S.A. (1996) 93(16):8455–9. doi: 10.1073/pnas.93.16.8455 PMC38692 8710892

[86] Rao AS Kremenevskaja N Resch J Brabant G. Lithium stimulates proliferation in cultured thyrocytes by activating wnt/beta-catenin signalling. Eur J Endocrinol (2005) 153(6):929–38. doi: 10.1530/eje.1.02038 16322400

[87] Baethge C Blumentritt H Berghofer A Bschor T Glenn T Adli M. Long-term lithium treatment and thyroid antibodies: a controlled study. J Psychiatry Neurosci (2005) 30(6):423–7. PMC1277025 16327876

[88] van Melick EJ Wilting I Meinders AE Egberts TC. Prevalence and determinants of thyroid disorders in elderly patients with affective disorders: Lithium and nonlithium patients. Am J Geriatr Psychiatry (2010) 18(5):395–403. doi: 10.1097/JGP.0b013e3181c6584e 20429083

[89] Waldman SA Park D. Myxedema coma associated with lithium therapy. Am J Med (1989) 87(3):355–6. doi: 10.1016/S0002-9343(89)80168-8 2773973

[90] Shopsin B Shenkman L Blum M Hollander CS. Iodine and lithium-induced hypothyroidism. documentation of synergism. Am J Med (1973) 55(5):695–9. doi: 10.1016/0002-9343(73)90193-9 4749208

[91] Lee S Chow CC Wing YK Shek CC. Thyroid abnormalities during chronic lithium treatment in Hong Kong Chinese: a controlled study. J Affect Disord (1992) 26(3):173–8. doi: 10.1016/0165-0327(92)90013-V 1460167

[92] Baltaci AK Mogulkoc R Belviranli M. L-thyroxine-induced hyperthyroidism affects elements and zinc in rats. Bratisl Lek Listy (2013) 114(3):125–8. doi: 10.4149/BLL_2013_027 23406177

[93] Maouche N Meskine D Alamir B Koceir EA. Trace elements profile is associated with insulin resistance syndrome and oxidative damage in thyroid disorders: Manganese and selenium interest in Algerian participants with dysthyroidism. J Trace Elem Med Biol (2015) 32:112–21. doi: 10.1016/j.jtemb.2015.07.002 26302919

[94] Zhang F Liu N Wang X Zhu L Chai Z. Study of trace elements in blood of thyroid disorder subjects before and after 131I therapy. Biol Trace Elem Res (2004) 97(2):125–34. doi: 10.1385/BTER:97:2:125 14985623

[95] Mittag J Behrends T Nordstrom K Anselmo J Vennstrom B Schomburg L. Serum copper as a novel biomarker for resistance to thyroid hormone. Biochem J (2012) 443(1):103–9. doi: 10.1042/BJ20111817 22220593

[96] Liu Y Liu S Mao J Piao S Qin J Peng S. Serum trace elements profile in graves’ disease patients with or without orbitopathy in northeast China. BioMed Res Int (2018) p:3029379. doi: 10.1155/2018/3029379 PMC5818896 29546054

[97] Kim MJ Kim SC Chung S Kim S Yoon JW Park YJ. Exploring the role of copper and selenium in the maintenance of normal thyroid function among healthy koreans. J Trace Elem Med Biol (2020) 61:126558. doi: 10.1016/j.jtemb.2020.126558 32480050

[98] Theophanides T Anastassopoulou J. Copper and carcinogenesis. Crit Rev Oncol Hematol (2002) 42(1):57–64. doi: 10.1016/S1040-8428(02)00007-0 11923068

[99] Blazewicz A Dolliver W Sivsammye S Deol A Randhawa R Orlicz-Szczesna G. Determination of cadmium, cobalt, copper, iron, manganese, and zinc in thyroid glands of patients with diagnosed nodular goitre using ion chromatography. J Chromatogr B Analyt Technol BioMed Life Sci (2010) 878(1):34–8. doi: 10.1016/j.jchromb.2009.11.014 19944657

[100] Kosova F Cetin B Akinci M Aslan S Seki A Pirhan Y. Serum copper levels in benign and malignant thyroid diseases. Bratisl Lek Listy (2012) 113(12):718–20. doi: 10.4149/BLL_2012_162 23173630

[101] Brady DC Crowe MS Turski ML Hobbs GA Yao X Chaikuad A. Copper is required for oncogenic BRAF signalling and tumorigenesis. Nature (2014) 509(7501):492–6. doi: 10.1038/nature13180 PMC4138975 24717435

[102] Rezaei M Javadmoosavi SY Mansouri B Azadi NA Mehrpour O Nakhaee S. Thyroid dysfunction: how concentration of toxic and essential elements contribute to risk of hypothyroidism, hyperthyroidism, and thyroid cancer. Environ Sci pollut Res Int (2019) 26(35):35787–96. doi: 10.1007/s11356-019-06632-7 31701424

[103] Schomburg L. Selenium, selenoproteins and the thyroid gland: interactions in health and disease. Nat Rev Endocrinol (2011) 8(3):160–71. doi: 10.1038/nrendo.2011.174 22009156

[104] Betsy A Binitha M Sarita S. Zinc deficiency associated with hypothyroidism: an overlooked cause of severe alopecia. Int J Trichol (2013) 5(1):40–2. doi: 10.4103/0974-7753.114714 PMC3746228 23960398

[105] Ertek S Cicero AF Caglar O Erdogan G. Relationship between serum zinc levels, thyroid hormones and thyroid volume following successful iodine supplementation. Hormones (Athens) (2010) 9(3):263–8. doi: 10.14310/horm.2002.1276 20688624

[106] Gumulec J Masarik M Adam V Eckschlager T Provaznik I Kizek R. Serum and tissue zinc in epithelial malignancies: A meta-analysis. PloS One (2014) 9(6):e99790. doi: 10.1371/journal.pone.0099790 24941118 PMC4062461

[107] Kudabayeva KI Koshmaganbetova GK Mickuviene N Skalnaya MG Tinkov AA Skalny AV. Hair trace elements are associated with increased thyroid volume in schoolchildren with goiter. Biol Trace Elem Res (2016) 174(2):261–6. doi: 10.1007/s12011-016-0711-6 27106540

[108] Baltaci AK Dundar TK Aksoy F Mogulkoc R. Changes in the serum levels of trace elements before and after the operation in thyroid cancer patients. Biol Trace Elem Res (2017) 175(1):57–64. doi: 10.1007/s12011-016-0768-2 27263537

[109] Al-Sayer H Mathew TC Asfar S Khourshed M Al-Bader A Behbehani A. Serum changes in trace elements during thyroid cancers. Mol Cell Biochem (2004) 260(1-2):1–5. doi: 10.1023/B:MCBI.0000026027.20680.c7 15228079

[110] Emami A Nazem MR Shekarriz R Hedayati M. Micronutrient status (calcium, zinc, vitamins d and e) in patients with medullary thyroid carcinoma: A cross-sectional study. Nutrition (2017) 41:86–9. doi: 10.1016/j.nut.2017.04.004 28760434

[111] Pathak R Pathak A. Effectiveness of zinc supplementation on lithium-induced alterations in thyroid functions. Biol Trace Elem Res (2021) 199(6):2266–71. doi: 10.1007/s12011-020-02356-9 32851540

[112] Soldin OP Aschner M. Effects of manganese on thyroid hormone homeostasis: potential links. Neurotoxicology (2007) 28(5):951–6. doi: 10.1016/j.neuro.2007.05.003 PMC2067987 17576015

[113] Reubi JC Chayvialle JA Franc B Cohen R Calmettes C Modigliani E. Somatostatin receptors and somatostatin content in medullary thyroid carcinomas. Lab Invest (1991) 64(4):567–73. 1673164

[114] Memon NS Kazi TG Afridi HI Baig JA Sahito OM Baloch S. Correlation of manganese with thyroid function in females having hypo- and hyperthyroid disorders. Biol Trace Elem Res (2015) 167(2):165–71. doi: 10.1007/s12011-015-0277-8 25774040

[115] Eder K Kralik A Kirchgessner M. The effect of manganese supply on thyroid hormone metabolism in the offspring of manganese-depleted dams. Biol Trace Elem Res (1996) 55(1-2):137–45. doi: 10.1007/BF02784175 8971361

[116] Kawada J Nishida M Yoshimura Y Yamashita K. Manganese ion as a goitrogen in the female mouse. Endocrinol Jpn (1985) 32(5):635–43. doi: 10.1507/endocrj1954.32.635 4092670

[117] Savchenko OV Toupeleev PA. Lead, cadmium, manganese, cobalt, zinc and copper levels in whole blood of urban teenagers with non-toxic diffuse goiter. Int J Environ Health Res (2012) 22(1):51–9. doi: 10.1080/09603123.2011.588324 21660794

[118] Stojsavljević A Trifković J Rasić-Milutinović Z Jovanović D Bogdanović G Mutić J. Determination of toxic and essential trace elements in serum of healthy and hypothyroid respondents by ICP-MS: A chemometric approach for discrimination of hypothyroidism. J Trace Elem Med Biol (2018) 48:134–40. doi: 10.1016/j.jtemb.2018.03.020 29773171

[119] Stojsavljević A Rovčanin B Krstić Đ Borković-Mitić S Paunović I Kodranov I. Evaluation of trace metals in thyroid tissues: Comparative analysis with benign and malignant thyroid diseases. Ecotoxicol Environ Saf (2019) 183:109479. doi: 10.1016/j.ecoenv.2019.109479 31365889

[120] Hsu JM Root AW Duckett GE Smith JC Jr. Yunice AA Kepford G. The effect of magnesium depletion on thyroid function in rats. J Nutr (1984) 114(8):1510–7. doi: 10.1093/jn/114.8.1510 6747732

[121] Liu M Song J Jiang Y Liu Y Peng J Liang H. A case-control study on the association of mineral elements exposure and thyroid tumor and goiter. Ecotoxicol Environ Saf (2021) 208:111615. doi: 10.1016/j.ecoenv.2020.111615 33396135

[122] van Gerwen M Alerte E Alsen M Little C Sinclair C Genden E. The role of heavy metals in thyroid cancer: A meta-analysis. J Trace Elem Med Biol (2022) 69:126900. doi: 10.1016/j.jtemb.2021.126900 34798515

[123] Assem FL Holmes P Levy LS. The mutagenicity and carcinogenicity of inorganic manganese compounds: a synthesis of the evidence. J Toxicol Environ Health B Crit Rev (2011) 14(8):537–70. doi: 10.1080/10937404.2011.615111 22008092

[124] Anastassopoulou J Theophanides T. Magnesium-DNA interactions and the possible relation of magnesium to carcinogenesis. irradiation and free radicals. Crit Rev Oncol Hematol (2002) 42(1):79–91. doi: 10.1016/S1040-8428(02)00006-9 11923070

[125] Szmeja Z Konczewska H. Red blood cell, serum and tissue magnesium levels in subjects with laryngeal carcinoma. ORL J Otorhinolaryngol Relat Spec (1983) 45(2):102–7. doi: 10.1159/000275631 6843967

[126] Leung PL Li XL. Multielement analysis in serum of thyroid cancer patients before and after a surgical operation. Biol Trace Elem Res (1996) 51(3):259–66. doi: 10.1007/BF02784080 8727673

[127] Durlach J Bara M Guiet-Bara A Collery P. Relationship between magnesium, cancer and carcinogenic or anticancer metals. Anticancer Res (1986) 6(6):1353–61. 3545048

[128] Jancic SA Stosic BZ. Cadmium effects on the thyroid gland. Vitam Horm (2014) 94:391–425. doi: 10.1016/B978-0-12-800095-3.00014-6 24388198

[129] Shen F Cai WS Li JL Feng Z Cao J Xu B. The association between serum levels of selenium, copper, and magnesium with thyroid cancer: a meta-analysis. Biol Trace Elem Res (2015) 167(2):225–35. doi: 10.1007/s12011-015-0304-9 25820485

[130] Ige AO Chidi RN Egbeluya EE Jubreel RO Adele BO Adewoye EO. Amelioration of thyroid dysfunction by magnesium in experimental diabetes may also prevent diabetes-induced renal impairment. Heliyon (2019) 5(5):e01660. doi: 10.1016/j.heliyon.2019.e01660 31193031 PMC6513790

[131] Digiesi V Bandinelli R Bisceglie P Santoro E. Magnesium in tumoral tissues, in the muscle and serum of subjects suffering from neoplasia. Biochem Med (1983) 29(3):360–3. doi: 10.1016/0006-2944(83)90071-6 6615492

[132] Pavia Junior MA Paier B Noli MI Hagmuller K Zaninovich AA. Evidence suggesting that cadmium induces a non-thyroidal illness syndrome in the rat. J Endocrinol (1997) 154(1):113–7. doi: 10.1677/joe.0.1540113 9246944

[133] Benvenga S Marini HR Micali A Freni J Pallio G Irrera N. Protective effects of myo-inositol and selenium on cadmium-induced thyroid toxicity in mice. Nutrients (2020) 12(5):1222. doi: 10.3390/nu12051222 PMC7282027 32357526

[134] Benvenga S Micali A Ieni A Antonelli A Fallahi P Pallio G. The association of myo-inositol and selenium contrasts cadmium-induced thyroid c cell hyperplasia and hypertrophy in mice. Front Endocrinol (Lausanne) (2021) 12:608697. doi: 10.3389/fendo.2021.608697 33716965 PMC7949001

[135] Zoeller RT Tan SW Tyl RW. General background on the hypothalamic-pituitary-thyroid (HPT) axis. Crit Rev Toxicol (2007) 37(1-2):11–53. doi: 10.1080/10408440601123446 17364704

[136] Buha A Matovic V Antonijevic B Bulat Z Curcic M Renieri EA. Overview of cadmium thyroid disrupting effects and mechanisms. Int J Mol Sci (2018) 19(5):1501. doi: 10.3390/ijms19051501 PMC5983752 29772829

[137] Luca E Fici L Ronchi A Marandino F Rossi ED Caristo ME. Intake of boron, cadmium, and molybdenum enhances rat thyroid cell transformation. J Exp Clin Cancer Res (2017) 36(1):73. doi: 10.1186/s13046-017-0543-z 28577555 PMC5455132

[138] Malandrino P Russo M Ronchi A Minoia C Cataldo D Regalbuto C. Increased thyroid cancer incidence in a basaltic volcanic area is associated with non-anthropogenic pollution and biocontamination. Endocrine (2016) 53(2):471–9. doi: 10.1007/s12020-015-0761-0 26438396

[139] Chung HK Russo M Ronchi A Minoia C Cataldo D Regalbuto C. Some elements in thyroid tissue are associated with more advanced stage of thyroid cancer in Korean women. Biol Trace Elem Res (2016) 171(1):54–62. doi: 10.1007/s12011-015-0502-5 26419761

[140] Faure R Dussault JH. Interaction of sodium molybdate with the thyroid hormone receptor. Biochem Cell Biol (1990) 68(3):630–4. doi: 10.1139/o90-089 2375853

[141] Yorita Christensen KL. Metals in blood and urine, and thyroid function among adults in the united states 2007-2008. Int J Hyg Environ Health (2013) 216(6):624–32. doi: 10.1016/j.ijheh.2012.08.005 23044211

[142] Çelik T Savaş N Kurtoğlu S Sangün Ö Aydın Z Mustafa D. Iodine, copper, zinc, selenium and molybdenum levels in children aged between 6 and 12 years in the rural area with iodine deficiency and in the city center without iodine deficiency in hatay. Turk Pediatri Ars (2014) 49(2):111–6. doi: 10.5152/tpa.2014.1209 PMC4462287 26078645

[143] Liang C Han Y Ma L Wu X Huang K Yan S. Low levels of arsenic exposure during pregnancy and maternal and neonatal thyroid hormone parameters: The determinants for these associations. Environ Int (2020) 145:106114. doi: 10.1016/j.envint.2020.106114 33035893

[144] Sun X Liu W Zhang B Shen X Hu C Chen X. Maternal heavy metal exposure, thyroid hormones, and birth outcomes: A prospective cohort study. J Clin Endocrinol Metab (2019) 104(11):5043–52. doi: 10.1210/jc.2018-02492 30994896

[145] Hughes MF. Arsenic toxicity and potential mechanisms of action. Toxicol Lett (2002) 133(1):1–16. doi: 10.1016/S0378-4274(02)00084-X 12076506

[146] Kim K Argos M Persky VW Freels S Sargis RM Turyk ME. Associations of exposure to metal and metal mixtures with thyroid hormones: Results from the NHANES 2007-2012. Environ Res (2022) 212(Pt C):113413. doi: 10.1016/j.envres.2022.113413 35537494

[147] Grimm D. Cell and molecular biology of thyroid disorders. Int J Mol Sci (2019) 20(12):1990. doi: 10.3390/ijms20122895 PMC6627965 31200596

[148] Sun HJ Xiang P Luo J Hong H Lin H Li HB. Mechanisms of arsenic disruption on gonadal, adrenal and thyroid endocrine systems in humans: A review. Environ Int (2016) 95:61–8. doi: 10.1016/j.envint.2016.07.020 27502899

[149] Correia MM Chammas MC Zavariz JD Arata A Martins LC Marui S. Evaluation of the effects of chronic occupational exposure to metallic mercury on the thyroid parenchyma and hormonal function. Int Arch Occup Environ Health (2020) 93(4):491–502. doi: 10.1007/s00420-019-01499-0 31832764

[150] Stojsavljevic A Rovcanin B Jagodic J Radojkovic DD Paunovic I Gavrovic-Jankulovic M. Significance of arsenic and lead in hashimoto’s thyroiditis demonstrated on thyroid tissue, blood, and urine samples. Environ Res (2020) 186:109538. doi: 10.1016/j.envres.2020.109538 32334172

[151] Gallagher CM Meliker JR. Mercury and thyroid autoantibodies in U.S. women, NHANES 2007-2008. Environ Int (2012) 40:39–43. doi: 10.1016/j.envint.2011.11.014 22280926

[152] Jain RB Choi YS. Interacting effects of selected trace and toxic metals on thyroid function. Int J Environ Health Res (2016) 26(1):75–91. doi: 10.1080/09603123.2015.1020416 25788177

